# Bat humoral immunity and its role in viral pathogenesis, transmission, and zoonosis

**DOI:** 10.3389/fimmu.2024.1269760

**Published:** 2024-08-02

**Authors:** Anne A. Roffler, Daniel P. Maurer, Tamika J. Lunn, Tarja Sironen, Kristian M. Forbes, Aaron G. Schmidt

**Affiliations:** ^1^ Ragon Institute of Mass General, MIT, and Harvard, Cambridge, MA, United States; ^2^ Department of Biological Sciences, University of Arkansas, Fayetteville, AR, United States; ^3^ Department of Virology, University of Helsinki, Helsinki, Finland; ^4^ Department of Veterinary Biosciences, University of Helsinki, Helsinki, Finland; ^5^ Department of Microbiology, Harvard Medical School, Boston, MA, United States

**Keywords:** Chiroptera, bat immunity, humoral responses, immunoglobulin repertoire, infectious diseases

## Abstract

Bats harbor viruses that can cause severe disease and death in humans including filoviruses (e.g., Ebola virus), henipaviruses (e.g., Hendra virus), and coronaviruses (e.g., SARS-CoV). Bats often tolerate these viruses without noticeable adverse immunological effects or succumbing to disease. Previous studies have largely focused on the role of the bat’s innate immune response to control viral pathogenesis, but little is known about bat adaptive immunity. A key component of adaptive immunity is the humoral response, comprised of antibodies that can specifically recognize viral antigens with high affinity. The antibody genes within the 1,400 known bat species are highly diverse, and these genetic differences help shape fundamental aspects of the antibody repertoire, including starting diversity and viral antigen recognition. Whether antibodies in bats protect, mediate viral clearance, and prevent transmission within bat populations is poorly defined. Furthermore, it is unclear how neutralizing activity and Fc-mediated effector functions contribute to bat immunity. Although bats have canonical Fc genes (e.g., mu, gamma, alpha, and epsilon), the copy number and sequences of their Fc genes differ from those of humans and mice. The function of bat antibodies targeting viral antigens has been speculated based on sequencing data and polyclonal sera, but functional and biochemical data of monoclonal antibodies are lacking. In this review, we summarize current knowledge of bat humoral immunity, including variation between species, their potential protective role(s) against viral transmission and replication, and address how these antibodies may contribute to population dynamics within bats communities. A deeper understanding of bat adaptive immunity will provide insight into immune control of transmission and replication for emerging viruses with the potential for zoonotic spillover.

## 1 Introduction

The immune system has innate and adaptive arms that have evolved to respond to a variety of diverse pathogens (1). Upon infection, the innate immune system rapidly responds through germline-encoded innate immune receptors that recognize common molecular patterns [e.g., Lipopolysaccharide (LPS), flagellin, and RNA] shared among many pathogens (1). In contrast, the adaptive immune system recognizes antigens unique to a pathogen (1) and can generate a “memory” response, which can protect from future infections by the same or similar pathogen, such as repeated exposures to viruses (1).

A major component of adaptive immunity is the humoral response, comprised of both B and T cells. Antibodies, produced by the former, have two functional domains: the antigen-binding fragment (Fab) that recognizes a specific epitope on an antigen and the crystallizable fragment (Fc) that drives effector functions (2, 3). Antibodies primarily recognize antigens with complementarity-determining regions (CDRs) on their Fab domain. Upon viral infection, naïve B cells that recognize the virus are selected to undergo somatic hypermutation, a mutational process known as affinity maturation, to refine specificity and increase affinity to the antigen (4, 5). In this way, adaptive immunity can generate antigen-specific, high-affinity antibodies that often neutralize viruses. Once bound to the virus, antibodies can interfere with key steps in the viral lifecycle. These can include sterically blocking the virus from binding to host cell receptors, preventing the virus from undergoing conformational changes required for fusion or entry, or inhibiting viral progeny release (6–9). The Fc region of bound antibodies also triggers a series of Fc-mediated responses that can activate innate immune cells (4).

**Figure 1:**
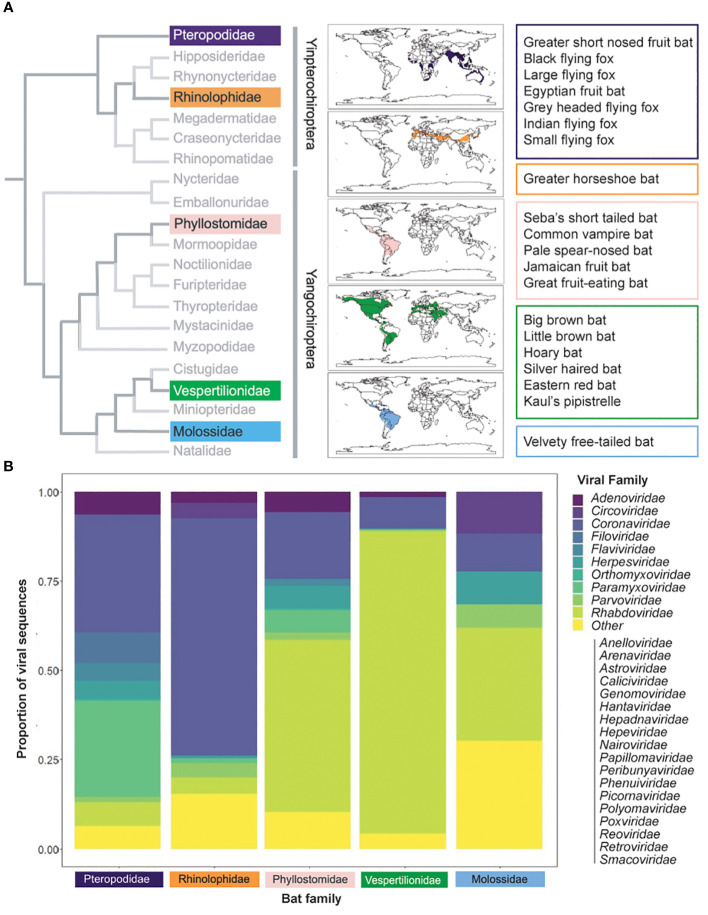
Bat species distribution and viral families. **(A)** Bats are diverse, with families that inhabit disparate regions across the globe. Calogram at the family level [adapated from (11)] at the family level. Colors correspond to families with named species in the text; named species are specified in colored boxes. Species distribution data from IUCN (2023) (12). **(B)** Bat viruses are similarly diverse. Species viral data from DBatVir (2023) (13). Proportions were calculated as the number of viruses (as per NCBI taxonomy) in each viral family per total number of viruses recorded for named species in the family.

Although the origin and evolution of humoral responses have been studied, the specific role and dynamics of antibody responses in bats are not well understood. Bats are a diverse taxa of mammals, second in species richness only to rodents, and are host to a wide range of pathogens (10) (**Figure 1A**). Their importance to public health have become increasingly recognized, as viruses that have caused severe outbreaks in humans have been found to circulate within bats, including paramyxoviruses, coronaviruses, and filoviruses (14–17) (**Figure 1B**). Several aspects of bat ecology and physiology are thought to make them particularly suitable to contract and maintain viruses, namely, their long lifespan, dense colonies, and multispecies roosts (10). Factors such as land use changes, wildlife hunting, and trade have increased human exposures to bats and their associated viruses (18, 19). As such, there is an urgent need to understand and mitigate the zoonotic hazards posed by viruses present in bats.

It is unclear how bat antibody responses impact viral replication, clearance, and persistence. This knowledge will help clarify infection dynamics at a population level and may help predict spillover risk. In this review, we summarize current knowledge of bat antibody immunity and its similarities and differences to mouse and human repertoires.

## 2 Antibody genes are diverse among bat species

**Figure 2:**
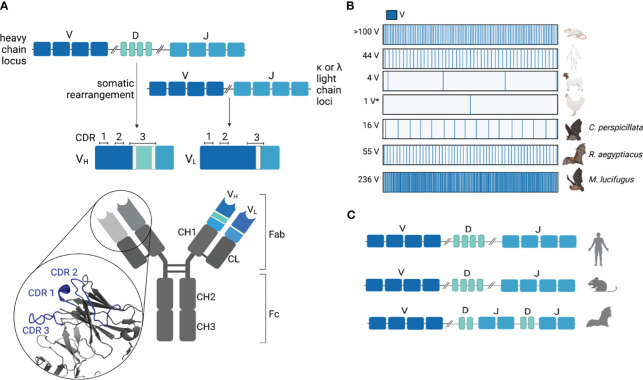
Immunoglobulin loci organization and gene abundance. **(A)** The variable region of antibodies is comprised of somatically rearranged V, D, and J (heavy chain) or V and J (light chain) genes. During somatic rearrangement, diversity is further introduced in the junctions between rearranged genes through the addition of non-templated P and N nucleotides (light green). The complementarity-determining regions (CDRs) are shown for both heavy and light chains. The heavy chain CDRs for antibody CH67 (PDB 4HKB) are highlighted in blue. **(B)** Functional V gene abundances are variable between species (*gene conversion). **(C)** Ig gene organization is unique for Egyptian rousette bats (ERBs) where D and J genes are interspersed rather than sequential (21), as seen in human and mouse Ig loci (22).

Antibodies are generated through the rearrangement of germline-encoded variable (V), diversity (D), and joining (J) immunoglobulin (Ig) gene segments (20) (**Figure 2A**). The antibody heavy chain variable region consists of rearranged V, D, and J gene segments, while the light chain consists of rearranged V and J segments only (23, 24). The variable region can be further divided into CDRs and framework regions (FWRs). The FWRs form, as their name suggests, the structural framework for the CDRs to protrude, like fingers, to interact with the antigen. Both the rearranged heavy and light chains have three flexible CDR loops, which largely determine antigen specificity (25).

Combinatorial diversity is introduced during B-cell development when V, D, and J segments in the heavy chain and the V and J segments in the light chain are rearranged to generate a pre-immune, or naïve, repertoire. Unlike CDR1 and CDR2, which are encoded by the V gene, CDR3 spans V, (D), and J genes. As such, gene rearrangement further diversifies the CDR3 through the addition of non-templated n nucleotides between gene segments. CDR3 is therefore the longest and the most variable of the CDRs, contributing significantly toward naïve repertoire diversity. The resulting naïve antibody is displayed on the surface of B cells as part of the B-cell receptor (BCR) complex. The genetic architecture of the Ig loci (i.e., location of gene segments, abundance of V genes, and sequence conservation between V gene segments) shapes the initial diversity of the naïve antibody repertoire. Although the antibody variable regions are further diversified through somatic mutation during B-cell maturation, baseline antigen specificity is defined by the germline Ig gene segments.

### 2.1 Number and sequence variability in bat immunoglobulin gene segments

The copy number of V, D, and J genes varies greatly between species (**Figure 2B**). The abundance of heavy chain V genes (V_H_) genes can range anywhere from >100 V_H_ (mice) and 44 V_H_ (humans) to four V_H_ (goats) (26, 27). Like mice, rabbits also have over 100 functional V_H_ genes but preferentially rearrange one gene to generate the majority of their antibody repertoire (28). In contrast, chickens rearrange a single functional V_H_ and numerous V_H_ pseudogenes to generate their naïve repertoire, a process known as somatic gene conversion (29). After a B cell recognizes an antigen through the BCR, rounds of somatic hypermutation (SHM) and affinity-based selections further refine the specificity defined by the germline usage and CDR3 compositions (4). This process is called affinity maturation.

Antibody genes are diverse between bat species. Early transcriptional and genomic sequencing from the big brown bat (*Eptesicus fuscus*) (30), Seba’s short-tailed bat (*Carollia perspicillata*) (30), greater short-nosed fruit bat (*Cynopterus sphinx*) (30), black flying fox (*Pteropus alecto*) (23), and large flying fox (*Pteropus vampyrus*) (23) identified putative functional V_H_ genes that are diverse in primary sequences. The little brown bat (*Myotis lucifugus*) has an estimated 236 unique V_H_ genes from the V_H_3 family alone, along with at least 13 J_H_ and numerous D_H_ genes, though there is yet no reference genome for this species (31). These Ig sequences were identified using a V_H_3 family-specific forward primer and porcine recombination signal sequence (RSS) reverse primer to amplify and sequence germline V_H_3 genes (31). A total of 75 unique sequences were identified, and based on the frequency at which each unique sequence was recovered more than once, a probabilistic model was used to estimate the total number of little brown bat V_H_3 genes (31). In contrast, the IGDectective algorithm has been leveraged to predict the Ig genes from four reference quality bat genomes (32), none of which seem to share the same expansion of V_H_ genes as the little brown bat (33). This algorithm identified 10 V_H_ from the velvety free-tailed bat, nine V_H_ from Kaul’s pipistrelle, 32 V_H_ from the pale spear-nosed bat (*Phyllostomus discolor*), and 63 V_H_ from the greater horseshoe bat (33). It is possible that IGDectective underestimated the number of V_H_ genes from these four bat species, as divergent RSS that do not pass the likelihood threshold of the algorithm are excluded from analysis (33). If bats do have a significant number of divergent RSS motifs, one would expect that a porcine RSS reverse primer would also undersample a significant portion of little brown bat V genes, unless divergent RSS motifs are highly bat species-specific. It is also possible that the probabilistic model used to estimate the number of little brown bat V_H_ genes did not capture the true quantity of V genes, as it relies on a relatively small sampling of 90 sequences (31).

The most thoroughly annotated bat Ig locus to date is that of the Egyptian rousette bat (ERB) (*Rousettus aegyptiacus*), providing the most detailed understanding of any bat Ig architecture (21). ERB has 66 V_H_ genes (55 functional, 10 pseudogenes, and one truncated), eight functional D_H_ genes, and nine J_H_ genes (seven functional and two pseudogenes) (21). Unlike other mammalian immunoglobulin heavy chain (IGH) loci where V, D, and J genes are organized sequentially, the ERB D and J genes are interspersed (i.e., IGHD-J-D-J) (21) (**Figure 2C**). When compared to the predicted Ig genes from the velvety free-tailed bat (*Molossus molossus*), Kaul’s pipistrelle (*Pipistrellus kuhlii*), and greater horseshoe bat (*Rhinolophus ferrumequinum*) with their sequential D to J organization, this interspersed organization is not shared between all bats (33). It is unclear how this heavy chain organization impacts recombination between V, D, and J segments.

The inter-species differences in V_H_ gene sequence and quantity suggest distinct encoded specificities of each species’ naïve antibody repertoire. Together, the abundance and primary sequence diversities of putative functional V_H_ genes between bat species suggest varying degrees of naïve repertoire diversity and specificities.

### 2.2 Anti-viral immunoglobulin gene expansion and germline biasing

Co-evolution with viruses can shape the diversity and specificity of the naïve repertoire (21, 34). Certain V genes are known to predispose antibodies toward recognizing specific antigens (35, 36). These V genes may have germline-associated features that have a functional effect, such as encoding CDR lengths or amino acids in fixed positions that facilitate binding (37, 38). V genes are under positive selection to maximize binding diversity while retaining specificity (34). This type of selection can present as the genomic expansion of V genes encoding antibodies with predispositions as well as the preferential usage of certain V genes upon infection. The former would be evident in germline V gene abundance, while the latter would require transcriptomic and proteomic analyses of the rearranged and expressed antibody repertoire. Precursor biasing can be leveraged to design vaccines that target naïve B-cell precursors from germlines known to elicit broadly neutralizing antibodies (39–41). It is unclear whether the bat Ig germlines have similarly been shaped to respond effectively against the viruses that circulate in them. ERBs, the reservoir for Marburg virus (MARV) (42), have expanded V_H_ genes that are associated with protective responses against viruses in humans. In particular, ERBs have expanded V_H_ genes associated with protection against Ebola virus (EBOV) (V_H_1–8, four copies; V_H_3–23, five copies; V_H_3–48, five copies) and MARV (V_H_4–59, eight copies; one copy each of V_H_4–61, V_H_4–39, and V_H_3–7) based on sequence similarity to human V genes (21). Although these expanded V genes suggest that ERBs could elicit a comparable protective response against MARV and EBOV, biochemical and biophysical characterization of isolated monoclonal antibodies is necessary to demonstrate that this is possible.

## 3 Affinity maturation and somatic hypermutation

Antigen specificity is largely defined by three CDRs on the heavy and light chains, which form the antigen combining site (34, 43, 44) (**Figure 2A**). CDR1 and CDR2 are germline encoded by the V_L_ and V_H_ elements of the light and heavy chains, respectively. CDR3 of the light chain is encoded primarily by the V segment, with additional diversity introduced at the V and J junctions during recombination. CDR3 of the heavy chain spans the end of the V gene to the beginning of the J gene, with substantial junctional diversity introduced somatically through V–DJ joining (34). The heavy chain CDR3 can therefore be variable in length and sequence identity. It is the key determinant for specificity in antigen recognition (44). While the germline V(D)J segments form the initial binding specificity of the naïve repertoire, subsequent rounds of SHM introduce mutations that improve affinity and refine specificity (45). Mutations introduced typically occur in the CDRs to directly influence binding, whereas mutations within framework regions can impact antibody folding and CDR conformation (46, 47).

### 3.1 Bat antibody CDR3 features

The CDR3 lengths of several bat species have been characterized based on Ig transcript sequences. Black flying fox CDR3 regions ranged from six to 18 amino acids (aa) in length, comparable to IgG transcript CDR3 lengths isolated from the little brown bat (4–12 aa), big brown bat (4–13 aa), Seba’s short-tailed bat (8–12 aa), and greater short-nosed fruit bat (4–12 aa) (23, 30).

The sequence compositions of CDR3 in mice and human repertoires are typically enriched for tyrosine, glycine, and serine (48). In the black flying fox, the CDR3 is de-enriched for tyrosine residues in germline D_H_ and J_H_ segments and in Ig transcripts (23). The cDNA transcripts of the Jamaican fruit bat (*Artibeus jamaicensis*) infected with Tacaribe virus (TCRV) demonstrate a large distribution of tyrosines within the CDR3, ranging from two tyrosine residues to six (49). Enrichment of tyrosine residues specifically for the Jamaican fruit bat could be indicative of affinity-matured antibodies against TCRV. Some arenaviruses, including TCRV, bind an exposed tyrosine residue on receptor TfR1 to mediate viral entry (50). There are human neutralizing antibodies against arenaviruses that bind the viral receptor binding pocket with tyrosines on their CDRs (50). It is possible that the enrichment of tyrosines in Jamaican fruit bats predisposes the repertoire to neutralize through the same pathway, though this is unlikely, as experimental infection with TCRV is typically fatal to Jamaican fruit bats, and only a few surviving bats had detectable neutralizing antibody titers (51). Without a reference genome, it is not possible to determine whether CDR3 tyrosine residues were germline encoded for this bat species, which could indicate germline biasing toward the generation of neutralizing antibodies.

### 3.2 Diversification through somatic hypermutation

SHM, mainly in the CDR regions, enhances diversity (52); indeed, SHM within the CDR3 can be sufficient to diversify the antibody repertoire of germline-restricted mice with only one functional V_H_ to recognize a multitude of unique antigens (44). The degree to which affinity maturation diversifies the bat antibody repertoire is not well characterized. One hypothesis is that the “accordion-like” expansion of V_H_3 genes in the little brown bat provides sufficient combinatorial diversity through recombination alone and that SHM, which was estimated to be low (31), is not essential in the little brown bat (31). It is also possible that the probabilistic model used overestimated the number of germline V genes. For bat species where germline V gene information is available, none share this “accordion” expansion of V_H_3 genes as inferred for the little brown bat. Indeed, transcriptomic studies of the Jamaican fruit bat found that activation-induced cytidine deaminase (AID), the protein involved in class switching and affinity maturation, was not induced after infection with TCRV (49, 53). Even without the apparent induction of AID, Jamaican fruit bats generated multiple class-switched antibody isotypes, a process that requires functional AID in other animals (49, 53). It is possible that the apparent lack of AID induction is due to the difficulty of evaluating enzyme transcripts in RNA-seq data with differential expression analysis. Although experimental infection of Jamaican fruit bats with TCRV proved mostly fatal, a minority of bats survived, with or without detectable neutralizing antibody titers (51). If AID was not induced in Jamaican fruit bats after infection, then it is unclear how bats were able to mount neutralizing antibodies, unless those neutralizing antibodies were germline encoded. In the black flying fox, nucleotide substitutions are present in germline and Ig transcript sequences indicative of SHM (23). However, without more comprehensive analysis of the transcribed repertoire and annotated bat Ig loci, it is difficult to accurately describe the degree of SHM that occurs during bat Ig maturation and whether reliance on SHM is species dependent.

## 4 Kappa and lambda light chain usage

The antibody variable light chain (V_L_) also contributes to antigen recognition by direct binding with light chain CDRs and/or by influencing the conformation of the heavy chain (54, 55). The light chain is encoded either by the kappa or lambda loci (56). The ratio of kappa to lambda antibodies varies between species ranging from 95:5 in mice, 60:40 in humans, to 7:93 in horses (26, 57). Birds have evolved to express only lambda light chains (58, 59).

There is evidence that bats generate antibodies with lambda light chains. For example, a mouse monoclonal antibody specific to big brown bat lambda light chain Igs cross-reacts with Yangochiroptera [e.g., little brown bat, hoary bat (*Lasiurus cinereus*), silver-haired bat (*Lasionycteris noctivagans*), and eastern red bat (*Lasiurus borealis*)] Igs but not with Yinpterochiroptera [e.g., grey-headed flying fox (*Pteropus poliocephalus*), large flying fox, and Indian flying fox (*Pteropus giganteus*)] Igs (60), suggesting that the lambda light chain sequence is conserved within Yangochiroptera, but Yinpterochiroptera have sequence-divergent light chains. No kappa light chains were detected in big brown bat serum using Protein L magnetic beads that bind kappa light chains from many, but not all, mammalian species (60). It is possible that the big brown bat expresses kappa light chains and that none were detected in this experiment due to the limited breadth of the Protein L beads. Both kappa and lambda light chain transcripts were identified in the black flying fox (Yinpterochiroptera) (61). Lambda transcripts were more abundant than kappa transcripts, which could indicate preferential lambda usage in Yinpterochiroptera. Several outstanding questions remain, including whether Yangochiroptera generates kappa light chain antibodies, the kappa:lambda usage ratio among bat species, and whether bats rearrange one light chain before the next similar to humans (62).

## 5 Antibody isotypes and subclasses

The Fc of an antibody bridges the adaptive and innate immune systems. The Fc domain engages receptors (FcR) on innate immune cells and exerts their effector functions (63). Humans have five antibody isotypes (IgG, IgM, IgD, IgA, and IgE), some of which are further differentiated into subclasses (e.g., IgG1, IgG2, IgG3, and IgG4). Each antibody isotype and when applicable, subclass are recognized by multiple host FcRs, each associated with its unique set of effector functions (64). The genes that encode the Fc are located in a cluster downstream of the J segments in the heavy chain locus (34). After rearrangement, B cells undergo class switching primarily before entering the germinal center reaction where affinity maturation of the variable regions takes place (65). The resultant antibody gains Fc-specific effector functions and retains antigen binding specificity.

Antibody isotypes are structurally distinct. For example, human IgM forms pentameric complexes that can avidly bind multiple antigens at once and activate complement-mediated clearance of infection (3). Due to their varied functions, each isotype is differentially expressed in each part of the body, and their expression levels are tightly regulated.

### 5.1 Immunoglobulin isotypes and expression profiles

Our understanding of bat antibody isotypes ranges from genomic sequences to transcriptomic data and characterizations of serum antibodies. IgG, IgA, IgE, and IgM isotypes have been identified both in the bat genome and transcriptionally (21, 30, 61). So far, IgD transcripts have only been identified in little brown bats and big brown bats, further illustrating divergent Ig repertoires among species and suborders (21, 30, 49, 61). Black flying fox IgG and IgM are abundant in the lymph node and spleen, consistent with expression patterns in ERBs (21). IgA is highly transcribed in the lungs of both black flying foxes and ERBs. Black flying fox brain, heart, and kidney tissues showed low expression of all three Ig isotypes, whereas ERBs have moderate expression of these isotypes in the same tissues (21, 66). Circulating antibodies in black flying foxes are predominantly IgG and IgM, with low levels of IgA detected (66).

In humans, IgG is the most abundant immunoglobulin isotype in serum and plays a direct role in controlling viral infections (67). IgG is similarly abundant in bat serum (68). There is evidence that bats transfer maternal IgG through both the placenta and mammary gland. IgG isolated from three species [long-fingered bat (*Myotis capaccinii*), greater mouse-eared bat (*Myotis myotis*), and common noctule (*Nyctalus noctule*)] were able to bind human and mouse neonatal Fc receptor (FcRn), the receptor responsible for transfer of maternal IgG across the placenta (69). Unlike other mammals, bat IgG was dominant over IgA in maternal lacteal secretion, suggesting an additional pathway to transfer maternal antibodies (66). Pups born to vaccinated or naturally infected, seropositive dams inherit maternal virus-specific antibodies (70). Maternal IgG is detectable for up to 3–5 months in ERB pups (71, 72) and up to 7.5 and 8.5 months in small flying foxes (*Pteropus hypomelanus*) and black flying foxes, respectively (70). Transfer of maternal antibody transfer has implications for population immunity and viral maintenance as bats typically have synchronized breeding and birthing cycles marked by the influx of colony size and routes of transmission followed by new pups that will lose their maternal antibodies within a year from birth (73).

The annotated ERB heavy chain locus is the highest resolution of the Fc segments of an important viral reservoir species. ERB IgA and IgM are predicted to share similar functions as their human counterparts. However, gene ontology analysis of ERB IgG and IgE predicts divergent functions compared to their human homologs (21).

### 5.2 Immunoglobulin subtypes are species specific

ERBs have an expanded set of epsilon genes, with two functional and three pseudogenes, making this species the only known mammal with two functional IgE subtypes (21). In humans, IgE binds to FcεRI expressed on the surface of mast cells and basophils. Though both IgE subtypes are expressed in circulation and secondary lymphoid organs in ERBs, only IgE2 is detectable in the lungs and bone marrow (21). Unlike human IgE, ERB IgE1 has an internal deletion compared to human IgE1 that shortens its cytosolic tail, potentially impacting downstream signaling (21). Tissue expression and structural differences between the two IgE subclasses in ERBs likely indicate divergent functions, though those functions are not yet known.

Several bat species seem to express multiple IgG and IgM subtypes. The ERB locus contains four gamma subtypes that have varying tissue expression profiles and differ primarily in the hinge and CH2 domains (21, 67). Black flying foxes also appear to have multiple subtypes of IgG and IgM (66), consistent with transcripts of multiple IgG subtypes identified in the little brown bat (five subtypes), greater short-nosed fruit bat (three subtypes), and big brown bat (two subtypes) (30). There are currently no functional data for the bat IgG subtypes or whether these subtypes share structural similarity with human IgG subtypes.

Several outstanding questions remain for bat Ig isotypes. The handful of species whose Fc compositions have been characterized show divergence between species. Although we now have more in-depth knowledge of ERB Fc sequences and tissue expression profiles (21), their unique Fc makeup is likely not widely applicable across all bat species. Speculation on function based on sequence alone requires further validation. It remains to be seen what the varying quantities of expressed Ig isotypes are among species, the affinity of each Ig isotype for their Fc receptors, the tissue expression profiles for each isotype paired with the detection of antibody in that tissue (to delineate tissue-specific expression and recruitment of antibodies to a tissue type from elsewhere), and, most importantly, detailed understanding of bat Fc effector functions. There is no reported functional characterization of bat isotypes, especially in the context of innate immune cells and complement activation, or the relative abundance of each isotype produced after infection or vaccination.

## 6 Biochemical and structural characteristics

Our understanding of bat antibodies is based almost entirely on sequencing and bulk serum analysis of antibody responses after infection. There is limited understanding of the biophysical characteristics of bat monoclonal antibodies, which appear to have unique structural properties. An N-linked glycosylation site at position 297 is conserved across ERB and human IgGs and is important for FcγR binding. Interestingly, ERB IgG1 contains a unique N-linked glycosylation motif at the hinge region, in addition to N297, that is not present in other ERB or human IgGs. Glycans can shield or cover important functional domains, restrict otherwise flexible protein domains, and affect protein folding. IgG1 is the most expressed antibody isotype and subtype in ERBs, and it is unknown how the N-linked glycan at the IgG1 hinge region may impact antibody folding, receptor binding, and effector function. Unlike IgG1, ERB IgG2, IgG3, and IgG4 all lack the canonical CxxC motif that enables inter-chain disulfide bonding in human IgGs (21). Without inter-chain disulfide bonding, it is possible that ERB IgG2, IgG3, and IgG4 can undergo Fab-arm exchange to become bispecific antibodies, a process observed for human IgG4 (74). For humans, Fab-arm exchange reduces immune activation as bispecific IgG4 cannot crosslink antigen (74). In contrast, the greater short-nosed fruit bat and the little brown bat IgG subtypes contain the canonical CxxC motif as identified through Ig transcript sequencing (30). Without structural and biochemical characterization of bat antibodies, one can only speculate on how the loss of CxxC impacts Ig folding, assembly, and the types of antibodies secreted.

ERB IgG2, IgG3, and IgG4 also have an amino acid substitution (Leu234Pro) in the lower hinge region that removes an important contact in human IgGs for Fc receptor FcγR (21). This substitution may impact affinity for FγR and subsequently impact effector functions downstream of binding [i.e., antibody-dependent cellular cytotoxicity, immune complex clearance, and phagocytosis of pathogens] (21). These sequencing differences could be indicative of biochemical and structural characteristics of bat antibodies that could further our understanding of anti-viral function and duration of responses.

Cysteine-rich V genes from velvety free-tailed bats, pale spear-nosed bats, and greater horseshoe bats can be found on the IMGT database (33). These V genes contain more than the two conserved cysteines that form a disulfide bond between frameworks 1 and 3 (33). They have additional cysteines in both their CDR1 and CDR2 loops (33). The ERB IGH locus similarly contains V_H_ family cysteines in both CDR1 and CDR2 loops (V_H_4–30 and V_H_4–39) (21). It is likely that the cysteines in CDR1 and CDR2 form an intrachain disulfide bond that would impact antibody conformation, hypervariable loop rigidity, and antigen specificity.

There is much to be learned from the biochemical and structural characterization of individual bat antibodies. These types of studies can uncover bat antibody binding affinities, viral epitope targeting, divergent antibody structural features, and more definitely quantify the neutralization profiles of viral-specific antibodies. Recombinant bat antibodies would also enable a more detailed analysis of Fc-mediated effector functions.

## 7 Antibody responses after infection

Several parameters determine the effectiveness of an antibody response. The first parameter is the magnitude and durability of the virus-specific primary and secondary responses (i.e., antibody titers) (75). The primary response occurs during the first viral encounter, where naïve B cells affinity-mature to improve specificity and affinity. Affinity-matured B cells can differentiate into plasma cells that circulate and secrete high-affinity antibodies or develop into memory B cells. The secondary response occurs upon re-exposure to the same or very similar virus. Unlike the primary response where high-affinity B cells are generated *de novo*, the secondary response is marked by rapid and robust antibody production as class-switched memory B cells already exist (76).

The abundance of neutralizing antibodies within the elicited antibody pool also influences viral clearance and transmission. Neutralizing antibodies directly prevent viruses from infecting host cells, by either blocking receptor engagement or viral fusion. The longevity of the memory compartment varies between species and against viruses, which determines the length of protection offered after primary exposure. For example, the yellow fever vaccine provides lifelong immunity with measurable IgG titers 40 years after a single vaccination, whereas annual influenza vaccinations are needed to maintain partial immunity (77). These temporal dynamics are shaped by both the durability of the antibody response and the mutability of the viral pathogen. Viruses that mutate frequently (e.g., influenza virus) can readily evade humoral memory responses.

### 7.1 Temporal dynamics of bat antibody responses

Bats generate antigen-specific antibodies upon immunization and natural or experimental infection. One of the first studies to demonstrate this was through experimental immunization of big brown bats with bacteriophage øX174 (78). Virus-specific bat antibodies have since been identified for EBOV (16), Japanese encephalitis virus (JEV) (79), MERS-CoV (80), Dengue virus (81), Nipah virus (NiV) (82, 83), Hendra virus (HeV) (83, 84), and MARV (71, 72, 85–88). Virus-specific antibodies are typically detectable between 10 and 28 days post-inoculation (dpi) (72, 82, 83, 85), with peak IgG levels measured at 14 dpi (71, 72). However, some studies report no seroconversion upon experimental infection with HeV (83), EBOV (89), and MERS-CoV (80). In humans, after primary infection, there is typically a lag where antigen-specific responses shift from IgM to IgG, accounting for B-cell class switching and generation of high-affinity secreted antibodies. This also appears to be the case for bats. For example, bats infected with JEV show a delayed shift from IgM to IgG response approximately 20 dpi (79).

The durability of the primary antibody response varies widely between bat species, infecting agents, and immune history. When inoculated with MARV, ERB antibody titers begin to wane as early as 30 dpi, falling below detection 3–4 months post-infection (71, 72, 85, 86). MARV-positive, wild-caught ERBs had detectable antibody titers up to 11 months post-capture, though their exposure history is unknown (72). This durability could be due to multiple re-exposures to MARV prior to capture, as re-exposure is known to maintain the memory B-cell compartment (90). Wild-caught common vampire bats (*Desmodus rotundus*), vaccinated and then challenged with rabies virus, similarly elicit neutralizing antibodies with variable timelines for decline, from as early as 43 days to 117 days (91). Virus-specific antibody responses in bats are reported to rapidly decline after infection with NiV (83), HeV (83), and JEV (92).

The magnitude of antibody responses in bats is generally lower compared to that of other mammals. Bats immunized with bacteriophage øX174 elicited a less robust response with lower anti-phage activity compared to guinea pigs and rabbits in the same study, despite receiving the same stimuli (78). An early study of bats immunized with sheep erythrocytes also found that bats generate fewer primary neutralizing antibody-secreting cells compared to mice (93). Specific pathogen-free Jamaican fruit bats had low IgG titers after SARS-CoV-2 infection with low levels of IL-21, which is expressed by T follicular helper cells that aid B cells undergoing affinity maturation (94). Jamaican fruit bats also generated low antibody titers against MERS-CoV (80), H18N11 influenza virus (95), and TCRV (51). These are surprising observations given the abundance of B cells in bat spleens and in circulation. Recent studies report that B cells make up roughly 30% of peripheral blood mononuclear cells and 35% of spleen cells from black flying foxes (96), comparable to mice B-cell abundance, and that 90% of B cells are IgG^+^ (97). There also appears to be an age-dependent fluctuation of B-cell quantity, where juvenile ERBs had approximately fourfold higher numbers of circulating B cells compared to their adult counterparts (98).

### 7.2 Efficacy of bat humoral immunity

A major outstanding question is whether antibody responses in bats are protective against viral infections, as measured by viral clearance and degree of viral transmission. Infected bats can shed their viruses through their bodily fluids (i.e., saliva, blood, and urine). Viral load in these samples is a proxy for transmission potential. For grey-headed flying foxes inoculated with NiV (82) or HeV (84), the presence of virus-specific antibodies coincided with viral clearance. This is consistent with black flying foxes inoculated with HeV, where the virus was cleared from blood and urine samples in bats with measurable antibody titers (83). A single grey-headed flying fox inoculated with NiV had detectable virus in urine samples despite the presence of neutralizing antibodies in circulation (82). Wild-caught great fruit-eating bats (*Artibeus intermedius*) experimentally infected with rabies virus rapidly mounted a neutralizing antibody response and were protected from disease (99), though the immune history of these bats is unknown.

There is extensive literature focusing on ERB antibody responses upon experimental infection with MARV. MARV-naïve ERBs seroconverted within 14 dpi and did not transmit MARV to co-housed naïve bats (71). Another study reports naïve ERBs seroconverted with peak MARV-specific antibodies between 14 and 28 dpi. All bats in this study became viremic between 5 and 12 dpi, with oral and rectal shedding detectable up until 14 dpi and 8 dpi, respectively (85). This is consistent with the report of Amman et al. that MARV is cleared from the blood by 10 dpi and that oral shedding peaks at 9 dpi and lasts up to 14 dpi (88). In these bats, MARV antibody titers are detectable by 9 dpi (88). In all three studies, MARV-specific antibody titers coincide with a decline in viral load (71, 85, 88). Naïve ERBs co-housed with infected ERBs did not develop detectable viremia and did not seroconvert (71). This suggests that the infected ERBs did not transmit their virus to their naïve housemates.

Typically, the abundance of neutralizing antibodies is a proxy for protection. Though virus-specific antibodies correlate with viral clearance in experimentally infected bats, the protective role neutralizing antibodies play in bats is still unclear. Serological surveillance of wild-caught bats from around the world has identified neutralizing antibodies against paramyxoviruses (e.g., NiV) (100), lyssaviruses (e.g., Lagos bat virus) (101), and flaviviruses (e.g., dengue virus) (81, 102). Bats experimentally infected with JEV generated 100-fold lower neutralizing titers compared to guinea pig counterparts (79). Low neutralizing titers were similarly observed for experimental infection with NiV (83), HeV (83), and MARV (72). Experimental prime and prime-boost regimes with EBOV or Sosuga virus did not elicit detectable neutralizing antibodies from ERBs (87). This is surprising due to the expanded V_H_ genes associated with protective responses against viruses that have been identified in ERBs (21). Despite low neutralizing titers, bats appear to retain short- and long-term humoral immunity upon re-infection with homologous virus (discussed in the next section) (72, 85, 86).

Experimental immunization and vaccination studies are direct measurements of protective humoral immunity. Experimental vaccination of wild-caught common vampire bats with replicating vaccinia virus expressing rabies glycoprotein elicits a short-term protective antibody response (103). Rabies virus is one of few viruses pathogenic and potentially lethal to bats. After vaccination, bats are protected between 18 and 90 days post-vaccination, with maximal protection 30 days after vaccination (80% survival) (103). Anti-rabies antibody titers drop by 120 days after vaccination, and bats are no longer protected upon challenge (103). The highest rate of seroconversion was observed 30 days after vaccination, where half of the vaccinated bats had anti-rabies antibodies (103). Interestingly, bats do not seroconvert by 18 days after vaccination but are still protected upon challenge with 60% survival (103). Seronegative bats vaccinated 30 or 90 days prior to the challenge are similarly protected in the absence of anti-rabies antibodies (103). It is worth noting that the bats used in this study were wild-caught, albeit from a region without documented cases of sylvatic rabies. Note that their immune histories are not known, as this could impact antibody responses.

A separate study similarly showed that wild-caught common vampire bats vaccinated with replicating vaccinia virus expressing rabies glycoprotein are protected from lethal challenge, even without detectable anti-rabies antibodies (104). In this study, all bats were challenged 31 days after vaccination. Of the 31 surviving vaccinated bats, nine did not have detectable anti-rabies antibodies at the time of lethal challenge (104). Sera collected from representative bats 90 days after the challenge contained high titers of anti-rabies antibodies (104). Wild-caught big brown bats vaccinated with raccoonpox (RCN) virus expressing either rabies glycoprotein or mosaic rabies glycoprotein did not consistently mount anti-rabies neutralizing antibodies, though both vaccinated groups showed improved survival against rabies challenge (91).

### 7.3 Memory compartment

Memory recall forms the basis of long-term humoral immunity (90). Upon re-exposure to a homologous or similar virus, high-affinity memory B cells are quickly reactivated and clonally expand. Bats immunized with bacteriophage øX174 generate neutralizing antibody titers that rapidly rise after the first and second boosts (78). The majority of neutralizing antibodies were initially 2-ME-sensitive IgM but transitioned to predominantly 2-ME-insensitive IgG by 28 dpi (78). As expected for animals with a memory compartment, the secondary antibody response inactivated phage more rapidly than those in the primary response (78).

ERBs have protective short- and long-term immunological memory to MARV. ERBs re-challenged with MARV generate a rapid secondary response as early as 5–10 dpi (71, 72). Rapid elicitation of virus-specific antibodies indicates a memory B-cell compartment. Seropositive ERBs challenged with MARV did not have viremia after 7 dpi, in contrast to their unprimed naïve counterparts (71). This suggests that immunological memory can offer short-term protection in ERBs against MARV re-challenge.

The majority of seropositive ERBs re-infected with a heterologous isolate of MARV became viremic despite a rapid secondary humoral response (72). ERB immunological memory to MARV may be relatively limited in breadth. Despite this, immunological memory seemed partially protective against heterologous challenge as evidenced by limited MARV replication in the spleen and liver of re-infected bats without systemic infection as seen in their naive counterparts (72).

The durability of the bat memory compartment is not fully characterized, with data exclusively for ERBs. Seronegative ERBs that had been previously infected with MARV 2 years prior mount robust MARV-specific antibodies as early as 7 days post-challenge with homologous virus (86). This robust secondary response was protective with no viral replication or shedding detectable (86), indicating that ERBs retain long-term immunological memory after primary infection.

Immunological memory has implications for transmission and viral maintenance in ERBs. In all three re-challenge studies, MARV was not detectable in any tissues that could contribute toward transmission (i.e., salivary glands, intestines, reproductive tract, and bladder) (71, 72), suggesting that MARV may require encounters with naïve bats or evolve to escape immunological memory for maintenance within the bat population.

A pitfall in these historical studies using wild-caught bats is that the immune history of these bats is unknown. Wild-caught bats without detectable antibody and virus titers at the time of capture are considered “naïve” in these studies, even though they could have been infected multiple times and their antibody titers have since waned. This complicates whether these bats are generating *de novo* antibody responses or memory recall upon a secondary exposure. It is likely that antibody responses of a truly naïve bat would be different from one that has sero-reverted, as the length of time between exposures as well as the number of exposures will impact the magnitude of the secondary response. The benefit of established bat colonies can remove some of these complicating factors, as the bats would have known immune histories. However, these colonies often pose financial and technical challenges, especially for insectivorous bats.

### 7.4 Immune control in bat populations

The durability and protectiveness of bat antibody responses form the basis for hypotheses around population immune control of viruses in bats. Three hypotheses dominate current research on population-level bat infection: that pulses of infection within populations are driven by 1) transmission of short-lived infections that provide long-lasting immunity [susceptible-infected-recovered (SIR) dynamics], 2) transmission of short-lived infections with fluctuating host immunity [susceptible-infected-recovered-susceptible (SIRS) dynamics], and 3) acute infection without clearance of virus, with subsequent transmission of reactivated viral infection [susceptible-infectious-latent-infectious (SILI) dynamics] (105). Within-host immune dynamics are a common driver among the three scenarios, but with distinct mechanisms to drive susceptibility and clearance of infection. Understanding the antibody response and its effects on current and future infections is a key challenge in deciphering these mechanisms. A better understanding of antibody responses will be key for interpretation and parameterization from prevalence and seroprevalence data.

## 8 Conclusions and future directions

Antibodies can engage viruses and their surface-exposed glycoproteins to directly neutralize or contribute to the clearance of infected cells through Fc-mediated functions. It is still unclear how such humoral immunity impacts viral pathogenesis, transmission, maintenance, and evolution in bat populations. While only a few bat species have been studied in-depth, their antibody genes and antibody responses vary greatly between species. This variation has directly contributed to observed differences in viral pathogenesis and transmission. Furthermore, studies involving wild-caught bats are complicated by unknown exposure histories. Establishing bat colonies would therefore enable controlled infection and vaccination studies to help deconvolute these complexities. Current studies on bat antibodies have largely been limited to sequence and transcriptomic data or serum analyses. Additional biochemical and biophysical characterization of the bat antibody repertoire including isolating monoclonal antibodies is needed. Such functional analyses would help uncover numerous features of bat humoral immunity, including epitope immunodominance, neautralization profiles, effector functions, and structural properties. This knowledge will greatly advance our understanding of host-pathogen interactions in an important viral reservoir and lay the groundwork for furthering our understanding of the bat immune system.

## Author contributions

AAR: Writing – original draft, Writing – review & editing. DM: Writing – review & editing. TJL: Writing – review & editing. KMF: Writing – review & editing, Funding acquisition. TS: Funding acquisition, Writing – review & editing. AGS: Funding acquisition, Writing – review & editing, Writing – original draft.

## References

[1] Dempsey PW Vaidya SA Cheng G. The art of war: Innate and adaptive immune responses. Cell Mol Life Sci. (2003) 60:2604–21. doi: 10.1007/s00018-003-3180-y PMC11138847 14685686

[2] Saphire EO Schendel SL Gunn BM Milligan JC Alter G. Antibody-mediated protection against Ebola virus. Nat Immunol. (2018) 19:1169–78. doi: 10.1038/s41590-018-0233-9 PMC6814399 30333617

[3] Lu LL Suscovich TJ Fortune SM Alter G. Beyond binding: antibody effector functions in infectious diseases. Nat Rev Immunol. (2018) 18:46–61. doi: 10.1038/nri.2017.106 29063907 PMC6369690

[4] Cyster JG Allen CDC. B cell responses: Cell interaction dynamics and decisions. Cell. (2019) 177:524–40. doi: 10.1016/j.cell.2019.03.016 PMC6538279 31002794

[5] Manz RA Thiel A Radbruch A. Lifetime of plasma cells in the bone marrow. Nature. (1997) 388:133–4. doi: 10.1038/40540 9217150

[6] Ge J Wang R Ju B Zhang Q Sun J Chen P. Antibody neutralization of SARS-CoV-2 through ACE2 receptor mimicry. Nat Commun. (2021) 12:250. doi: 10.1038/s41467-020-20501-9 33431856 PMC7801515

[7] Chen Y Wang F Yin L Jiang H Lu X Bi Y. Structural basis for a human broadly neutralizing influenza A hemagglutinin stem-specific antibody including H17/18 subtypes. Nat Commun. (2022) 13:7603. doi: 10.1038/s41467-022-35236-y 36494358 PMC9734383

[8] Dufloo J Planchais C Frémont S Lorin V Guivel-Benhassine F Stefic K. Broadly neutralizing anti-HIV-1 antibodies tether viral particles at the surface of infected cells. Nat Commun. (2022) 13:630. doi: 10.1038/s41467-022-28307-7 35110562 PMC8810770

[9] Kajihara M Marzi A Nakayama E Noda T Kuroda M Manzoor R. Inhibition of Marburg virus budding by nonneutralizing antibodies to the envelope glycoprotein. J Virol. (2012) 86:13467–74. doi: 10.1128/JVI.01896-12 PMC3503067 23035224

[10] Luis AD Hayman DTS O’Shea TJ Cryan PM Gilbert AT Pulliam JRC. A comparison of bats and rodents as reservoirs of zoonotic viruses: are bats special? Proc Biol Sci. (2013) 280:20122753. doi: 10.1098/rspb.2012.2753 23378666 PMC3574368

[11] Sadier A Urban DJ Anthwal N Howenstine AO Sinha I Sears KE. Making a bat: The developmental basis of bat evolution. Genet Mol Biol. (2021) 43:e20190146. 33576369 10.1590/1678-4685-GMB-2019-0146 PMC7879332

[12] IUCN Red List of Threatened Species. Version 3 (2023). Available online at: https://www.iucnredlist.org.

[13] DBatVir the Database of Bat-associated Viruses (2023). Available online at: http://www.mgc.ac.cn/cgi-bin/DBatVir/main.cgi. 10.1093/database/bau021 PMC3958617 24647629

[14] Towner JS Amman BR Sealy TK Carroll SAR Comer JA Kemp A. Isolation of genetically diverse Marburg viruses from Egyptian fruit bats. PLoS Pathog. (2009) 5:e1000536. doi: 10.1371/journal.ppat.1000536 19649327 PMC2713404

[15] Amman BR Albariño CG Bird BH Nyakarahuka L Sealy TK Balinandi S. A recently discovered pathogenic Paramyxovirus, sosuga virus, is present in Rousettus aEgyptiacus fruit bats at multiple locations in Uganda. J Wildl Dis. (2015) 51:774–9. doi: 10.7589/2015-02-044 PMC5022529 25919464

[16] Leroy EM Kumulungui B Pourrut X Rouquet P Hassanin A Yaba P. Fruit bats as reservoirs of Ebola virus. Nature. (2005) 438:575–6. doi: 10.1038/438575a 16319873

[17] Letko M Seifert SN Olival KJ Plowright RK Munster VJ. Bat-borne virus diversity, spillover and emergence. Nat Rev Microbiol. (2020) 18:461–71. doi: 10.1038/s41579-020-0394-z PMC7289071 32528128

[18] Plowright RK Eby P Hudson PJ Smith IL Westcott D Bryden WL. Ecological dynamics of emerging bat virus spillover. Proc Biol Sci. (2015) 282:20142124. doi: 10.1098/rspb.2014.2124 25392474 PMC4262174

[19] Ruiz-Aravena M McKee C Gamble A Lunn T Morris A Snedden CE. Ecology, evolution and spillover of coronaviruses from bats. Nat Rev Microbiol. (2022) 20:299–314. doi: 10.1038/s41579-021-00652-2 34799704 PMC8603903

[20] Tonegawa S. Somatic generation of antibody diversity. Nature. (1983) 302:575–81. doi: 10.1038/302575a0 6300689

[21] Larson PA Bartlett ML Garcia K Chitty J Balkema-Buschmann A Towner J. Genomic features of humoral immunity support tolerance model in Egyptian rousette bats. Cell Rep. (2021) 35:109140. doi: 10.1016/j.celrep.2021.109140 34010652

[22] Created with Biorender.com.

[23] Baker ML Tachedjian M Wang L-F. Immunoglobulin heavy chain diversity in Pteropid bats: evidence for a diverse and highly specific antigen binding repertoire. Immunogenetics. (2010) 62:173–84. doi: 10.1007/s00251-010-0425-4 PMC2887692 20162414

[24] Alt FW Blackwell TK Yancopoulos GD. Development of the primary antibody repertoire. Science. (1987) 238:1079–87. doi: 10.1126/science.3317825 3317825

[25] Jones PT Dear PH Foote J Neuberger MS Winter G. Replacing the complementarity-determining regions in a human antibody with those from a mouse. Nature. (1986) 321:522–5. doi: 10.1038/321522a0 3713831

[26] Sinkora M Stepanova K Butler JE Sinkora M Jr Sinkora S Sinkorova J. Comparative aspects of immunoglobulin gene rearrangement arrays in different species. Front Immunol. (2022) 13:823145. doi: 10.3389/fimmu.2022.823145 35222402 PMC8873125

[27] Das S Nozawa M Klein J Nei M. Evolutionary dynamics of the immunoglobulin heavy chain variable region genes in vertebrates. Immunogenetics. (2008) 60:47–55. doi: 10.1007/s00251-007-0270-2 18196235 PMC2386433

[28] Knight KL Becker RS. Molecular basis of the allelic inheritance of rabbit immunoglobulin VH allotypes: implications for the generation of antibody diversity. Cell. (1990) 60:963–70. doi: 10.1016/0092-8674(90)90344-E 2317867

[29] Reynaud CA Dahan A Anquez V Weill JC. Somatic hyperconversion diversifies the single Vh gene of the chicken with a high incidence in the D region. Cell. (1989) 59:171–83. doi: 10.1016/0092-8674(89)90879-9 2507167

[30] Butler JE Wertz N Zhao Y Zhang S Bao Y Bratsch S. The two suborders of chiropterans have the canonical heavy-chain immunoglobulin (Ig) gene repertoire of eutherian mammals. Dev Comp Immunol. (2011) 35:273–84. doi: 10.1016/j.dci.2010.08.011 20816694

[31] Bratsch S Wertz N Chaloner K Kunz TH Butler JE. The little brown bat, M. lucifugus, displays a highly diverse V H, D H and J H repertoire but little evidence of somatic hypermutation. Dev Comp Immunol. (2011) 35:421–30. doi: 10.1016/j.dci.2010.06.004 20547175

[32] Jebb D Huang Z Pippel M Hughes GM Lavrichenko K Devanna P. Six reference-quality genomes reveal evolution of bat adaptations. Nature. (2020) 583:578–84. doi: 10.1038/s41586-020-2486-3 PMC8075899 32699395

[33] Sirupurapu V Safonova Y Pevzner PA. Gene prediction in the immunoglobulin loci. Genome Res. (2022) 32:1152–69. doi: 10.1101/gr.276676.122 PMC9248892 35545447

[34] Rajewsky K Förster I Cumano A. Evolutionary and somatic selection of the antibody repertoire in the mouse. Science. (1987) 238:1088–94. doi: 10.1126/science.3317826 3317826

[35] Kirkham PM Mortari F Newton JA Schroeder HW Jr. Immunoglobulin VH clan and family identity predicts variable domain structure and may influence antigen binding. EMBO J. (1992) 11:603–9. doi: 10.1002/embj.1992.11.issue-2 PMC556492 1537339

[36] Chen F Tzarum N Wilson IA Law M. VH1-69 antiviral broadly neutralizing antibodies: genetics, structures, and relevance to rational vaccine design. Curr Opin Virol. (2019) 34:149–59. doi: 10.1016/j.coviro.2019.02.004 PMC7266006 30884330

[37] Pennell M Rodriguez OL Watson CT Greiff V. The evolutionary and functional significance of germline immunoglobulin gene variation. Trends Immunol. (2023) 44:7–21. doi: 10.1016/j.it.2022.11.001 36470826

[38] Thomson CA Bryson S McLean GR Creagh AL Pai EF Schrader JW. Germline V-genes sculpt the binding site of a family of antibodies neutralizing human cytomegalovirus. EMBO J. (2008) 27:2592–602. doi: 10.1038/emboj.2008.179 PMC2567409 18772881

[39] Briney B Sok D Jardine JG Kulp DW Skog P Menis S. Tailored immunogens direct affinity maturation toward HIV neutralizing antibodies. Cell. (2016) 166:1459–70.e11. doi: 10.1016/j.cell.2016.08.005 27610570 PMC5018249

[40] Jardine JG Kulp DW Havenar-Daughton C Sarkar A Briney B Sok D. HIV-1 broadly neutralizing antibody precursor B cells revealed by germline-targeting immunogen. Science. (2016) 351:1458–63. doi: 10.1126/science.aad9195 PMC4872700 27013733

[41] Stamatatos L Pancera M McGuire AT. Germline-targeting immunogens. Immunol Rev. (2017) 275:203–16. doi: 10.1111/imr.12483 PMC5741082 28133796

[42] Towner JS Pourrut X Albariño CG Nkogue CN Bird BH Grard G. Marburg virus infection detected in a common African bat. PLoS One. (2007) 2:e764. doi: 10.1371/journal.pone.0000764 17712412 PMC1942080

[43] Poljak RJ Amzel LM Chen BL Phizackerley RP Saul F. Structural basis for the association of heavy and light chains and the relation of subgroups to the conformation of the active site of immunoglobulins. Immunogenetics. (1975) 2:393–4. doi: 10.1007/BF01572309

[44] Xu JL Davis MM. Diversity in the CDR3 region of V(H) is sufficient for most antibody specificities. Immunity. (2000) 13:37–45. doi: 10.1016/S1074-7613(00)00006-6 10933393

[45] Tas JMJ Mesin L Pasqual G Targ S Jacobsen JT Mano YM. Visualizing antibody affinity maturation in germinal centers. Science. (2016) 351:1048–54. doi: 10.1126/science.aad3439 PMC4938154 26912368

[46] Julian MC Li L Garde S Wilen R Tessier PM. Efficient affinity maturation of antibody variable domains requires co-selection of compensatory mutations to maintain thermodynamic stability. Sci Rep. (2017) 7:45259. doi: 10.1038/srep45259 28349921 PMC5368667

[47] Koenig P Lee CV Walters BT Janakiraman V Stinson J Patapoff TW. Mutational landscape of antibody variable domains reveals a switch modulating the interdomain conformational dynamics and antigen binding. Proc Natl Acad Sci U S A. (2017) 114:E486–95. doi: 10.1073/pnas.1613231114 PMC5278476 28057863

[48] Zemlin M Klinger M Link J Zemlin C Bauer K Engler JA. Expressed murine and human CDR-H3 intervals of equal length exhibit distinct repertoires that differ in their amino acid composition and predicted range of structures. J Mol Biol. (2003) 334:733–49. doi: 10.1016/j.jmb.2003.10.007 14636599

[49] Gerrard DL Hawkinson A Sherman T Modahl CM Hume G Campbell CL. Transcriptomic signatures of Tacaribe virus-infected Jamaican fruit bats. mSphere. (2017) 2:e00245-17. doi: 10.1128/mSphere.00245-17 28959737 PMC5615131

[50] Mahmutovic S Clark L Levis SC Briggiler AM Enria DA Harrison SC. Molecular basis for antibody-mediated neutralization of new world hemorrhagic fever mammarenaviruses. Cell Host Microbe. (2016) 19:424. doi: 10.1016/j.chom.2016.02.008 PMC4685251 26651946

[51] Cogswell-Hawkinson A Bowen R James S Gardiner D Calisher CH Adams R. Tacaribe virus causes fatal infection of an ostensible reservoir host, the Jamaican fruit bat. J Virol. (2012) 86:5791–9. doi: 10.1128/JVI.00201-12 PMC3347293 22379103

[52] Berens SJ Wylie DE Lopez OJ. Use of a single VH family and long CDR3s in the variable region of cattle Ig heavy chains. Int Immunol. (1997) 9:189–99. doi: 10.1093/intimm/9.1.189 9043960

[53] David Q Schountz T Schwemmle M Ciminski K. Different but not unique: Deciphering the immunity of the Jamaican fruit bat by studying its viriome. Viruses. (2022) 14:238. doi: 10.3390/v14020238 35215832 PMC8879847

[54] Xiao H Guo T Yang M Qi J Huang C Hong Y. Light chain modulates heavy chain conformation to change protection profile of monoclonal antibodies against influenza A viruses. Cell Discov. (2019) 5:21. doi: 10.1038/s41421-019-0086-x 30993000 PMC6465249

[55] Sela-Culang I Kunik V Ofran Y. The structural basis of antibody-antigen recognition. Front Immunol. (2013) 4:302. doi: 10.3389/fimmu.2013.00302 24115948 PMC3792396

[56] van der Kant R Bauer J Karow-Zwick AR Kube S Garidel P Blech M. Adaption of human antibody λ and κ light chain architectures to CDR repertoires. Protein Eng Des Sel. (2019) 32:109–27. doi: 10.1093/protein/gzz012 PMC6908821 31535139

[57] Popov AV Zou X Xian J Nicholson IC Brüggemann M. A human immunoglobulin lambda locus is similarly well expressed in mice and humans. J Exp Med. (1999) 189:1611–20. doi: 10.1084/jem.189.10.1611 PMC2193639 10330440

[58] Das S Nikolaidis N Klein J Nei M. Evolutionary redefinition of immunoglobulin light chain isotypes in tetrapods using molecular markers. Proc Natl Acad Sci U S A. (2008) 105:16647–52. doi: 10.1073/pnas.0808800105 PMC2575474 18940927

[59] Das S Hirano M Tako R McCallister C Nikolaidis N. Evolutionary genomics of immunoglobulin-encoding Loci in vertebrates. Curr Genomics. (2012) 13:95–102. doi: 10.2174/138920212799860652 23024601 PMC3308330

[60] Lee WT Jones DD Yates JL Winslow GM Davis AD Rudd RJ. Identification of secreted and membrane-bound bat immunoglobulin using a Microchiropteran-specific mouse monoclonal antibody. Dev Comp Immunol. (2016) 65:114–23. doi: 10.1016/j.dci.2016.06.024 PMC7172696 27377583

[61] Papenfuss AT Baker ML Feng Z-P Tachedjian M Crameri G Cowled C. The immune gene repertoire of an important viral reservoir, the Australian black flying fox. BMC Genomics. (2012) 13:261. doi: 10.1186/1471-2164-13-261 22716473 PMC3436859

[62] Butler JE Wertz N Baker ML. The immunoglobulin genes of bats. In: Kaushil AK Pasman Y, editors. Comparative Immunoglobulin Genetics. Toronto, ON: Apple Academic Press (2014). p. 53–84.

[63] Mikocziova I Greiff V Sollid LM. Immunoglobulin germline gene variation and its impact on human disease. Genes Immun. (2021) 22:205–17. doi: 10.1038/s41435-021-00145-5 PMC8234759 34175903

[64] Spiegelberg HL. Biological role of different antibody classes. Int Arch Allergy Appl Immunol. (1989) 90(Suppl 1):22–7. doi: 10.1159/000235071 2693365

[65] Roco JA Mesin L Binder SC Nefzger C Gonzalez-Figueroa P Canete PF. Class-switch recombination occurs infrequently in germinal centers. Immunity. (2019) 51:337–50.e7. doi: 10.1016/j.immuni.2019.07.001 31375460 PMC6914312

[66] Wynne JW Di Rubbo A Shiell BJ Beddome G Cowled C Peck GR. Purification and characterisation of immunoglobulins from the Australian black flying fox (Pteropus alecto) using anti-fab affinity chromatography reveals the low abundance of IgA. PLoS One. (2013) 8:e52930. doi: 10.1371/journal.pone.0052930 23308125 PMC3538733

[67] Vidarsson G Dekkers G Rispens T. IgG subclasses and allotypes: from structure to effector functions. Front Immunol. (2014) 5:520. doi: 10.3389/fimmu.2014.00520 25368619 PMC4202688

[68] Chakraborty AK Chakravarty AK. Plaque forming cell assay for antibody secreting cells in the bat Pteropus giganteus. Indian J Exp Biol. (1983) 21:5–7. 6354927

[69] Toshkova N Zhelyazkova V Justesen S Dimitrov JD. Conservative pattern of interaction of bat and human IgG antibodies with FcRn. Dev Comp Immunol. (2023) 139:104579. doi: 10.1016/j.dci.2022.104579 36272453

[70] Epstein JH Baker ML Zambrana-Torrelio C Middleton D Barr JA DuBovi E. Duration of maternal antibodies against canine distemper virus and Hendra virus in pteropid bats. PLoS One. (2013) 8:e67584. doi: 10.1371/journal.pone.0067584 23826322 PMC3695084

[71] Paweska JT Jansen van Vuren P Fenton KA Graves K Grobbelaar AA Moolla N. Lack of Marburg virus transmission from experimentally infected to susceptible in-contact Egyptian fruit bats. J Infect Dis. (2015) 212 Suppl 2:S109–18. doi: 10.1093/infdis/jiv132 25838270

[72] Storm N Jansen Van Vuren P Markotter W Paweska J. Antibody responses to Marburg virus in Egyptian rousette bats and their role in protection against infection. Viruses. (2018) 10:73. doi: 10.3390/v10020073 29439384 PMC5850380

[73] Epstein JH Anthony SJ Islam A Kilpatrick AM Ali Khan S Balkey MD. Nipah virus dynamics in bats and implications for spillover to humans. Proc Natl Acad Sci U S A. (2020) 117:29190–201. doi: 10.1073/pnas.2000429117 PMC7682340 33139552

[74] Rispens T Huijbers MG. The unique properties of IgG4 and its roles in health and disease. Nat Rev Immunol. (2023) 23:763–78. doi: 10.1038/s41577-023-00871-z PMC10123589 37095254

[75] Röltgen K Powell AE Wirz OF Stevens BA Hogan CA Najeeb J. Defining the features and duration of antibody responses to SARS-CoV-2 infection associated with disease severity and outcome. Sci Immunol. (2020) 5:eabe0240. doi: 10.1126/sciimmunol.abe0240 33288645 PMC7857392

[76] Ademokun AA Dunn-Walters D. Immune responses: Primary and secondary. In: Encyclopedia of Life Sciences. John Wiley & Sons, Ltd. (2010) 9:526–35. doi: 10.1038/npg.els.0000947

[77] Pulendran B. Learning immunology from the yellow fever vaccine: innate immunity to systems vaccinology. Nat Rev Immunol. (2009) 9:741–7. doi: 10.1038/nri2629 19763148

[78] Hatten BA Allen R Sulkin SE. Immune response in Chiroptera to bacteriophage øX174. J Immunol. (1968) 101:141–50. doi: 10.4049/jimmunol.101.1.141 4874094

[79] Leonard LL Allen R Sulkin SE. Bat immunoglobulins formed in response to experimental Japan- ese B encephalitis (JBE) virus infection. J Immunol. (1968) 101:1168–75. doi: 10.4049/jimmunol.101.6.1168 4177527

[80] Munster VJ Adney DR van Doremalen N Brown VR Miazgowicz KL Milne-Price S. Replication and shedding of MERS-CoV in Jamaican fruit bats (Artibeus jamaicensis). Sci Rep. (2016) 6:21878. doi: 10.1038/srep21878 26899616 PMC4761889

[81] Platt KB Mangiafico JA Rocha OJ Zaldivar ME Mora J Trueba G. Detection of dengue virus neutralizing antibodies in bats from Costa Rica and Ecuador. J Med Entomol. (2000) 37:965–7. doi: 10.1603/0022-2585-37.6.965 11126559

[82] Middleton DJ Morrissy CJ van der Heide BM Russell GM Braun MA Westbury HA. Experimental Nipah virus infection in pteropid bats (Pteropus poliocephalus). J Comp Pathol. (2007) 136:266–72. doi: 10.1016/j.jcpa.2007.03.002 17498518

[83] Halpin K Hyatt AD Fogarty R Middleton D Bingham J Epstein JH. Pteropid bats are confirmed as the reservoir hosts of henipaviruses: a comprehensive experimental study of virus transmission. Am J Trop Med Hyg. (2011) 85:946–51. doi: 10.4269/ajtmh.2011.10-0567 PMC3205647 22049055

[84] Williamson MM Hooper PT Selleck PW Westbury HA Slocombe RF. Experimental hendra virus infectionin pregnant Guinea-pigs and fruit Bats (Pteropus poliocephalus). J Comp Pathol. (2000) 122:201–7. doi: 10.1053/jcpa.1999.0364 10684689

[85] Schuh AJ Amman BR Jones MEB Sealy TK Uebelhoer LS Spengler JR. Modelling filovirus maintenance in nature by experimental transmission of Marburg virus between Egyptian rousette bats. Nat Commun. (2017) 8:14446. doi: 10.1038/ncomms14446 28194016 PMC5316840

[86] Schuh AJ Amman BR Sealy TK Spengler JR Nichol ST Towner JS. Egyptian rousette bats maintain long-term protective immunity against Marburg virus infection despite diminished antibody levels. Sci Rep. (2017) 7:8763. doi: 10.1038/s41598-017-07824-2 28821722 PMC5562751

[87] Schuh AJ Amman BR Sealy TK Kainulainen MH Chakrabarti AK Guerrero LW. Antibody-mediated virus neutralization is not a universal mechanism of Marburg, Ebola, or Sosuga virus clearance in Egyptian rousette bats. J Infect Dis. (2019) 219:1716–21. doi: 10.1093/infdis/jiy733 PMC6519049 30590775

[88] Amman BR Jones MEB Sealy TK Uebelhoer LS Schuh AJ Bird BH. Oral shedding of Marburg virus in experimentally infected Egyptian fruit bats (Rousettus aEgyptiacus). J Wildl Dis. (2015) 51:113–24. doi: 10.7589/2014-08-198 PMC5022530 25375951

[89] Swanepoel R Leman PA Burt FJ Zachariades NA Braack LE Ksiazek TG. Experimental inoculation of plants and animals with Ebola virus. Emerg Infect Dis. (1996) 2:321–5. doi: 10.3201/eid0204.960407 PMC2639914 8969248

[90] Gourley TS Wherry EJ Masopust D Ahmed R. Generation and maintenance of immunological memory. Semin Immunol. (2004) 16:323–33. doi: 10.1016/j.smim.2004.08.013 15528077

[91] Cárdenas-Canales EM Velasco-Villa A Ellison JA Satheshkumar PS Osorio JE Rocke TE. A recombinant rabies vaccine that prevents viral shedding in rabid common vampire bats (Desmodus rotundus). PLoS Negl Trop Dis. (2022) 16:e0010699. doi: 10.1371/journal.pntd.0010699 36026522 PMC9455887

[92] van den Hurk AF Smith CS Field HE Smith IL Northill JA Taylor CT. Transmission of Japanese Encephalitis virus from the black flying fox, Pteropus alecto, to Culex annulirostris mosquitoes, despite the absence of detectable viremia. Am J Trop Med Hyg. (2009) 81:457–62. doi: 10.4269/ajtmh.2009.81.457 19706915

[93] Chakraborty AK Chakravarty AK. Antibody-mediated immune response in the bat, Pteropus giganteus. Dev Comp Immunol. (1984) 8:415–23. doi: 10.1016/0145-305X(84)90048-X 6376191

[94] Burke B Rocha SM Zhan S Eckley M Reasoner C Addetia A. Regulatory T cell-like response to SARS-CoV-2 in Jamaican fruit bats (Artibeus jamaicensis) transduced with human ACE2. PLoS Pathog. (2023) 19:e1011728. doi: 10.1101/2023.02.13.528205 37856551 PMC10617724

[95] Ciminski K Ran W Gorka M Lee J Malmlov A Schinköthe J. Bat influenza viruses transmit among bats but are poorly adapted to non-bat species. Nat Microbiol. (2019) 4:2298–309. doi: 10.1038/s41564-019-0556-9 PMC7758811 31527796

[96] Periasamy P Hutchinson PE Chen J Bonne I Shahul Hameed SS Selvam P. Studies on B cells in the fruit-eating black flying fox (Pteropus alecto). Front Immunol. (2019) 10:489. doi: 10.3389/fimmu.2019.00489 30930908 PMC6428034

[97] Martínez Gómez JM Periasamy P Dutertre C-A Irving AT Ng JHJ Crameri G. Phenotypic and functional characterization of the major lymphocyte populations in the fruit-eating bat Pteropus alecto. Sci Rep. (2016) 6:37796. doi: 10.1038/srep37796 27883085 PMC5121612

[98] Friedrichs V Toussaint C Schäfer A Rissmann M Dietrich O Mettenleiter TC. Landscape and age dynamics of immune cells in the Egyptian rousette bat. Cell Rep. (2022) 40:111305. doi: 10.1016/j.celrep.2022.111305 36070695

[99] Obregón-Morales C Aguilar-Setién Á Perea Martínez L Galvez-Romero G Martínez-Martínez FO Aréchiga-Ceballos N. Experimental infection of Artibeus intermedius with a vampire bat rabies virus. Comp Immunol Microbiol Infect Dis. (2017) 52:43–7. doi: 10.1016/j.cimid.2017.05.008 28673461

[100] Yob JM Field H Rashdi AM Morrissy C van der Heide B Rota P. Nipah virus infection in bats (Order chiroptera) in peninsular Malaysia. Emerg Infect Dis. (2001) 7:439–41. doi: 10.3201/eid0703.017312 PMC2631791 11384522

[101] Wright E Hayman DTS Vaughan A Temperton NJ Wood JLN Cunningham AA. Virus neutralising activity of African fruit bat (Eidolon helvum) sera against emerging lyssaviruses. Virology. (2010) 408:183–9. doi: 10.1016/j.virol.2010.09.014 PMC7172354 20951400

[102] Kading RC Kityo RM Mossel EC Borland EM Nakayiki T Nalikka B. Neutralizing antibodies against flaviviruses, Babanki virus, and Rift Valley fever virus in Ugandan bats. Infect Ecol Epidemiol. (2018) 8:1439215. doi: 10.1080/20008686.2018.1439215 29511459 PMC5827769

[103] Sétien AA Brochier B Tordo N De Paz O Desmettre P Péharpré D. Experimental rabies infection and oral vaccination in vampire bats (Desmodus rotundus). Vaccine. (1998) 16:1122–6. doi: 10.1016/S0264-410X(98)80108-4 9682368

[104] Aguilar-Setién A Leon YC Tesoro EC Kretschmer R Brochier B Pastoret P-P. Vaccination of vampire bats using recombinant vaccinia-rabies virus. J Wildl Dis. (2002) 38:539–44. doi: 10.7589/0090-3558-38.3.539 12243138

[105] Plowright RK Peel AJ Streicker DG Gilbert AT McCallum H Wood J. Transmission or within-host dynamics driving pulses of zoonotic viruses in reservoir-host populations. PLoS Negl Trop Dis. (2016) 10:e0004796. doi: 10.1371/journal.pntd.0004796 27489944 PMC4973921

